# The extent of violence inflicted on adolescent Aboriginal girls in the Northern Territory

**DOI:** 10.1186/s12889-022-13982-4

**Published:** 2022-08-29

**Authors:** Susan Moore, John R. Condon, Vincent YF He, Kylie Stothers, Tamika Williams, Steven Guthridge

**Affiliations:** 1 Centre for Child Development and Education, Menzies School of Health Research, Charles Darwin University, 41096, Building Red 9, Charles Darwin University, Casuarina campus, Ellengowan Drive., Casuarina, NT 0811 Australia; 2 Indigenous Allied Health Australia, Katherine, Northern Territory Australia

**Keywords:** Aboriginal and Torres Strait islander people, Adolescent women, Assault hospitalization, Child protective services, Physical and sexual abuse, Violence

## Abstract

**Background:**

Australian Aboriginal and Torres Strait Islander women are at very high risk of violence but there is little evidence about the age at which their higher exposure to violence commences. The aim of this study was to investigate violence inflicted on Aboriginal girls during childhood and adolescence, relative to Aboriginal boys and non-Aboriginal girls.

**Methods:**

This was a retrospective cohort study using de-identified administrative data for NT residents aged 0-17 years. This study used linked hospital and child protection data to investigate hospitalization for injury caused by assault and substantiated child maltreatment involving violence (physical and sexual abuse).

**Results:**

The incidence of assault hospitalization and substantiated physical/sexual abuse was much higher for Aboriginal than non-Aboriginal adolescents but similar for girls and boys to about age ten, then increased much more for Aboriginal girls than boys. In the 14-17 age-group, assault hospitalization incidence was 125% higher for Aboriginal girls than boys but 56% lower for non-Aboriginal girls than boys. 4.6% of Aboriginal girls were hospitalized (30.9% more than once) for assault between twelfth and eighteenth birthdays, compared to 3.4% of Aboriginal boys and 0.3% of non-Aboriginal girls. The incidence of assault hospitalization during adolescence was over three times higher for Aboriginal children who had substantiated child maltreatment during childhood.

**Conclusion:**

The very high levels of violence suffered by Aboriginal women commence in the pre-teen years. Non-Aboriginal girls are ‘protected’ from the rising levels of violence that boys experience as they progress through adolescence, but Aboriginal girls are not afforded such protection.

## Background

Aboriginal and Torres Strait Islander (hereafter respectfully referred to as Aboriginal) women are subject to much greater levels of violence, including sexual violence, than other Australian women [1]. In Australia, Aboriginal women are five times more likely than non-Aboriginal women to be victims of homicide by a current or former partner [2]. Nationally, hospitalization for injury is more common for Aboriginal than non-Aboriginal Australians but is considerably less common for females than males for both the Aboriginal and non-Aboriginal populations. However, the incidence of hospitalization for injury caused by assault is 30-50 times higher for Aboriginal than for non-Aboriginal women in early and middle adulthood [3]..

The pattern of hospitalization for injury is similar for children. For injury as a whole, incidence is higher for Aboriginal than non-Aboriginal children but lower for females than males in both populations [3]. Likewise, the incidence of hospitalization for injury caused by assault is much higher for Aboriginal than non-Aboriginal children (four to eight times higher for boys, depending on age-group, and nine to eighteen times higher for girls), but after 4 years of age assault hospitalization is more common for girls than boys in the Aboriginal population but much less common for girls than boys in the non-Aboriginal population [3]..

Violence experienced by Aboriginal children cannot be considered in isolation of the sociocultural and historic context of Australia’s colonialization [4] and its impact on children, families and communities that continue to the present day [5]. Lateral violence is recognized within Australia [5] and internationally [6] as behaviors occurring between First Nations peoples that encompasses gossip, bullying and physical violence or aggression within groups who are systemically exploited. Where families are unable to protect children, or where lateral violence [7] is normative within the kinship network or community as the result of intergenerational racial oppression, it can be difficult to determine what strategies, systems and services can most effectively support sustained behavior change that promotes the safety of children through to adulthood.

In the Northern Territory (NT), where Aboriginal people comprise 30% of the population, there has been particular focus on child sexual abuse which has been reported as being ‘serious, widespread and often unreported’ [8]. However, there is limited reliable evidence about the extent of physical and sexual violence inflicted on adolescent Aboriginal girls, and its consequences. Hospital admission rates of 13-17 year olds for conditions “indicative” of physical abuse have been reported as 13.5 times greater for Aboriginal than non-Aboriginal girls with a trend of increase across the period from 1999 to 2010 [9], while the incidence of hospitalization after assault has only been reported separately for sex and broad age groups [10]. Recent data is not available for Aboriginal boys and girls in the NT. Circumstantial evidence that may indicate high occurrence of sexual assault includes the very high rates of teenage pregnancy; in 2018, the proportion of first-time mothers aged less than 20 years was 2% for non-Aboriginal women but 42% for Aboriginal women (including 4% aged less than 16 years) [11]..

The particular vulnerability of adolescent girls in First Nations populations has been highlighted in international literature, such as in Canada [12]. In Australia there are frameworks [4, 13] to eliminate violence against children and violence against women, however the processes for protection of adolescent girls are unclear. Adolescent girls may be classified as either ‘children’ or ‘women’, but initiatives to support children fail to recognize the differences between boys and girls while programs for women fail to recognize the specific vulnerability of adolescent girls. As a consequence adolescent girls “remain invisible and fall through the cracks” [14].

This study aimed to investigate the extent of physical and sexual violence for adolescent Aboriginal girls and the relationship between early child maltreatment and risk of assault during the teenage years (the terms adolescent “boys” and “girls” are used throughout the manuscript for consistency with the wider literature, however the authors acknowledge, from a cultural context, many adolescent Aboriginal males and females are considered men and women). The paper considers the extent of violence inflicted on adolescent Aboriginal girls, with the intent that increased understanding and focus on their experience (within the broader context of the violence experienced by Aboriginal women) can lead to renewed emphasis on strategies that support safety, early intervention and prevention.

## Methods

### Study design and study population

This was a retrospective study using individual-level linked records for hospital admissions, substantiated child protection notifications and perinatal information to measure the levels of violence suffered by adolescents in the NT, comparing Aboriginal girls with Aboriginal boys and non-Aboriginal girls and boys. Two indicators of violence were used: hospitalization for assault (compared with hospitalization for all injuries); and substantiated physical or sexual abuse (compared with substantiated emotional abuse or neglect).

The study had three components. The first component investigated the age during childhood at which the level of violence experienced by Aboriginal girls diverged from Aboriginal boys and non-Aboriginal girls, by calculating age-specific incidence rates by single year of age for the period 2011-2016 by sex and Indigenous status for: hospitalization for any injury and for injury caused by assault; substantiated physical or sexual abuse and substantiated emotional abuse or neglect.

The second component investigated the proportion of Aboriginal adolescent girls (compared with Aboriginal boys and non-Aboriginal girls) who experienced violence between their twelfth and eighteenth birthdays by calculating the cumulative incidence of any assault hospitalization and any physical/sexual abuse, and the proportion of those with any event who experienced more than one such event, for a cohort of NT adolescents. The cohort consisted of NT residents who turned twelve between 1/1/2006 and 31/12/2010 (and so turned 18 before 31/12/2016) and had at least one record in any linked data repository dataset (see below) both before the age of 12 years and at or after the age of 17 years (which indicated that they had probably lived in the NT for the entire study period).

The third component investigated the association between maltreatment during childhood (before twelfth birthday) with assault hospitalization between twelfth and eighteenth birthdays for a cohort of children born in the NT in the period 1999-2003 (ascertained from the Perinatal Data Collection, see below) and who had at least one record in any of the repository datasets before the age of 12 years and at or after the age of 17 years.

### Data sources

Data for this study was obtained from an extensive repository of linked administrative data established by the Child and Youth Development Research Partnership (CYDRP), a collaboration between the Menzies School of Health Research and NT Government agencies. The CYDRP data repository contains de-identified unit-level information for NT children for 14 datasets extracted from NT Government administrative, statutory and service delivery data sources [15]. Individual linkage keys were prepared by SA-NT DataLink using probabilistic linkage with clerical review for uncertain matches [16]. Three key datasets were used for the study: the Hospital Inpatient dataset, which contain a summary of all hospital admissions in the five NT public hospitals; the Child Protection dataset which contains a record of all contacts with child protection services including all substantiated episodes of maltreatment; and the Perinatal Data Collection, a statutory register containing information about all births that occur in the NT. Other datasets in the repository were used to ascertain continuing NT residence of the study population.

An injury hospitalization was an inpatient episode with a primary or secondary diagnosis code in the International Classification of Diseases and Related Health Problems, 10th Revision, Australian Modification (ICD-10-AM) range S00-T75 or T79, which are those injuries usually sustained in the community setting (i.e. excluding hospitalizations for treatment of longer-term consequences of injury and injuries arising from medical treatment) [17]. Injury hospitalizations with an external cause coding of X85-Y09 were classified as assault hospitalizations. Contiguous inpatient episodes were combined as a single episode, such as inter-hospital transfers, statistical discharge and readmission at the same time, or self-discharge and re-admission within 1 day.

A substantiated episode of child maltreatment was a notification of possible maltreatment reported to child protection services where investigation assessed that there was sufficient reason to believe the child has been, is being, or is likely to be abused, neglected, or otherwise harmed. Maltreatment was classified as one or more of four types: physical abuse; sexual abuse, including sexual exploitation; emotional abuse, including exposure to family violence; and neglect [18]. When more than one maltreatment type was substantiated, the primary maltreatment type was the one which posed the greatest immediate risk to the child; other types of co-occurring maltreatment were recorded as ‘secondary types’. A substantiated episode of physical/sexual abuse was an episode in the Child Protection dataset with either a primary or secondary maltreatment type of physical and/or sexual abuse; similarly, an episode of emotional abuse/neglect was an episode with either as a primary or secondary maltreatment type.

### Statistical analysis

The age-specific incidence rate was calculated as the number of events (injury hospitalization; assault hospitalization; substantiated physical or sexual abuse; substantiated emotional abuse or neglect) that occurred between 1/1/2011 and 31/12/2016 for children aged 0-17 years at the time of occurrence divided by the corresponding Estimated Resident Population summed over the 5 years 2011-2016, stratified by single year of age, sex and Indigenous status.

Cumulative incidence was calculated as the proportion (expressed as a percentage) of NT residents who turned twelve years of age between 1/1/2006 and 31/12/2010 who had each event (and separately, two or more such events) between their twelfth and eighteenth birthdays.

Since 2002, ICD-10-AM assault codes have included a fifth-digit code for the relationship of the perpetrator of the assault to the victim; when there is more than one perpetrator, the closest familial connection is coded [19]. For assault hospitalizations between 2011 and 2016, we calculated the frequency distribution (excluding records with missing/unknown perpetrator data) of perpetrator relationship by Indigenous status, age-group and sex.

Multivariable Cox regression analysis was used to investigate the relationship between child protection involvement before twelfth birthday and assault hospitalization during adolescence (between twelfth and eighteenth birthday). This analysis was restricted to Aboriginal children because the number of non-Aboriginal adolescents with child protection contact before twelfth birthday and assault hospitalization after twelfth birthday was low. Survival time was defined as the time in years from the twelfth birthday to the occurrence of first injury/assault hospitalization, with follow-up time censored at eighteenth birthday. Variables included in the final model were: sex, female compared with male; and level of child protection contact before twelfth birthday, classified in four mutually exclusive categories: ‘No contact’ with child protection services (the reference category); ‘Notification only’, one or more notifications but no substantiations; ‘Substantiation only’, one or more substantiations but no out-of-home care placements; and ‘Out-of-home care’, at least one substantiation leading to out-of-home care placement. Analyses were conducted using Stata version 15.

## Results

### Divergence of girls from boys

**Fig. 1:**
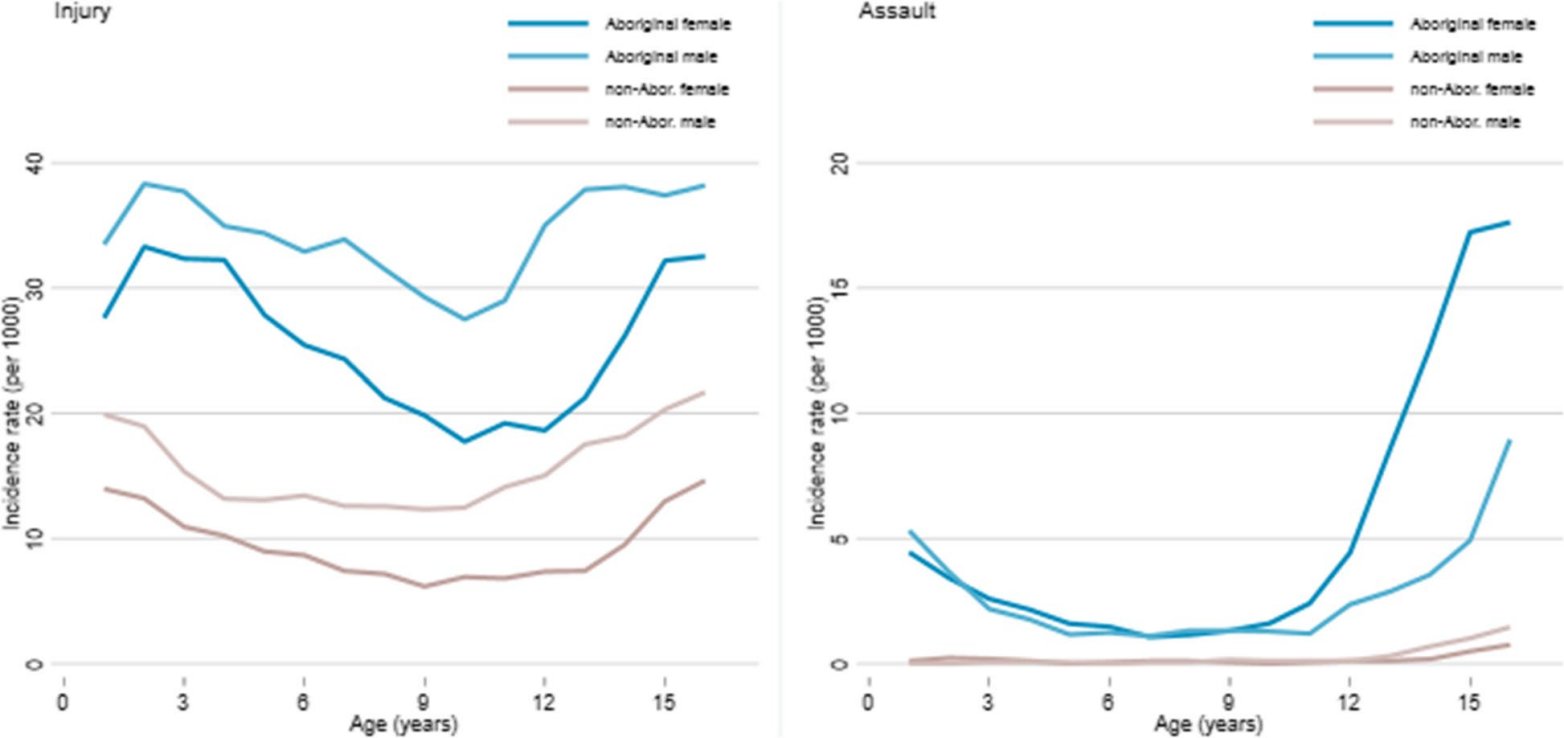
Incidence of hospitalization for treatment of injury and for treatment of injury caused by assault, by year of age,^1^ NT 2011-2016. 1. Moving three-year age-groups from 0 to 2 years old to 15-17 years old. X-axis is the middle year of each three-year age group

For all injury, the incidence of hospitalization was much higher for Aboriginal than non-Aboriginal children and considerably higher for boys than girls (both Aboriginal and non-Aboriginal) at all ages (Fig. 1). For injuries caused by assault, the incidence of hospitalization was much higher for Aboriginal than non-Aboriginal children (the incidence rate was close to zero for non-Aboriginal children until about age 14) and was similar for boys and girls in early and middle childhood (Table 1). For non-Aboriginal boys, incidence increased from about age 14; there was only a small increase for girls. For Aboriginal children, incidence increased from about age 12 (earlier for girls than boys) and the increase was much greater for girls than boys. For 14-17 year-old Aboriginal girls, 44% of injury hospitalizations were the result of assault (compared to 17.6% for Aboriginal boys and only 3.8% for non-Aboriginal girls) (Table 2) and the incidence of assault hospitalization was more than double that for Aboriginal boys and over 30 times that for non-Aboriginal girls (IRR 30.9, 95%CI 19.2-49.5).

**Table 1:** Assault hospitalization incidence rate^a^ by age-group, Northern Territory 2011-2016

Age-group	Aboriginal				Non-Aboriginal^c^			
	Male	Female	Ratio^b^		Male	Female	Ratio	
0-9	2.17	2.21	1.02	(0.74-1.38)	na	na	na	
10-13	2.09	3.56	1.70	(1.11-2.62)	na	na	na	
14-17	7.44	16.70	2.25	(1.80-2.80)	1.24	0.54	0.44	(0.22-0.88)

^a^ incidence rate per 1000 person-years

^b^ incidence rate ratio (female:male) and 95% confidence interval

^c^ for non-Aboriginal children the rate ratio was calculated only for the 14-17 year age-group because there were only 13 assault hospitalizations for non-Aboriginal children aged less than 14 years

na. Not available because of very small number of events for these categories

**Table 2:** Proportion (%) of injury hospitalizations that were caused by assault, NT 2011-2016

Age-group	Aboriginal		Non-Aboriginal	
	Male	Female	Male	Female
0-9	5.4	6.9	0.2	0.9
10-13	5.5	15.4	1.0	1.2
14-17	17.6	44.0	5.4	3.8

For Aboriginal children aged 0-9 years, a parent or carer was the perpetrator of over 70% of assaults that caused hospitalization (for which the perpetrator was recorded in hospital data), but this proportion was much lower for older children, less than 10% for those aged 14-17 years (Table 3). For 14-17 year olds, perpetrators of assault against girls were most commonly spouses or partners (40%) while for boys perpetrators were most commonly non-family members (47%). For non-Aboriginal adolescents aged 14-17 years the distribution was consistent with the pattern for Aboriginal males and females, although the number of assault hospitalizations with perpetrator recorded was small (only 18 in the 14-17 age-group, and 13 in the 0-9 and 10-13 age-groups combined).

**Table 3:** Perpetrators of assault: proportion of assault hospitalizations by category of perpetrator, NT 2011-2016

	Aboriginal						non-Aboriginal ^a^	
	Male			Female			Male	Female
	0-9	10-13	14-17	0-9	10-13	14-17	14-17	14-17
Number of assault hospitalizations (n)	83	34	120	77	52	241	29	11
Number with perpetrator recorded (n)	73	27	79	66	37	195	9	9
Proportion by perpetrator (%) ^b^								
Spouse or domestic partner	0.0	0.0	7.6	1.5^d^	16.2	39.5	0.0	44.4
Parent or carer	74.0	22.2	3.8	71.2	10.8	7.2	0.0	11.1
Other family member	16.4	22.2	41.8	19.7	40.5	28.7	0.0	0.0
Non-family ^c^	9.6	55.6	46.8	7.6	32.4	24.6	100.0	44.4

^a^ data is not presented for non-Aboriginal children aged 0-13 years because there were only 13 assault hospitalizations

^b^ proportion of hospitalizations with perpetrator recorded

^c^ friend, acquaintance, other specified, official authorities or unknown person(s)

^d^ one female aged 0-9 years had the perpetrator recorded as a ‘spouse or domestic partner’; it is not known whether this was a data entry error

**Fig. 2:**
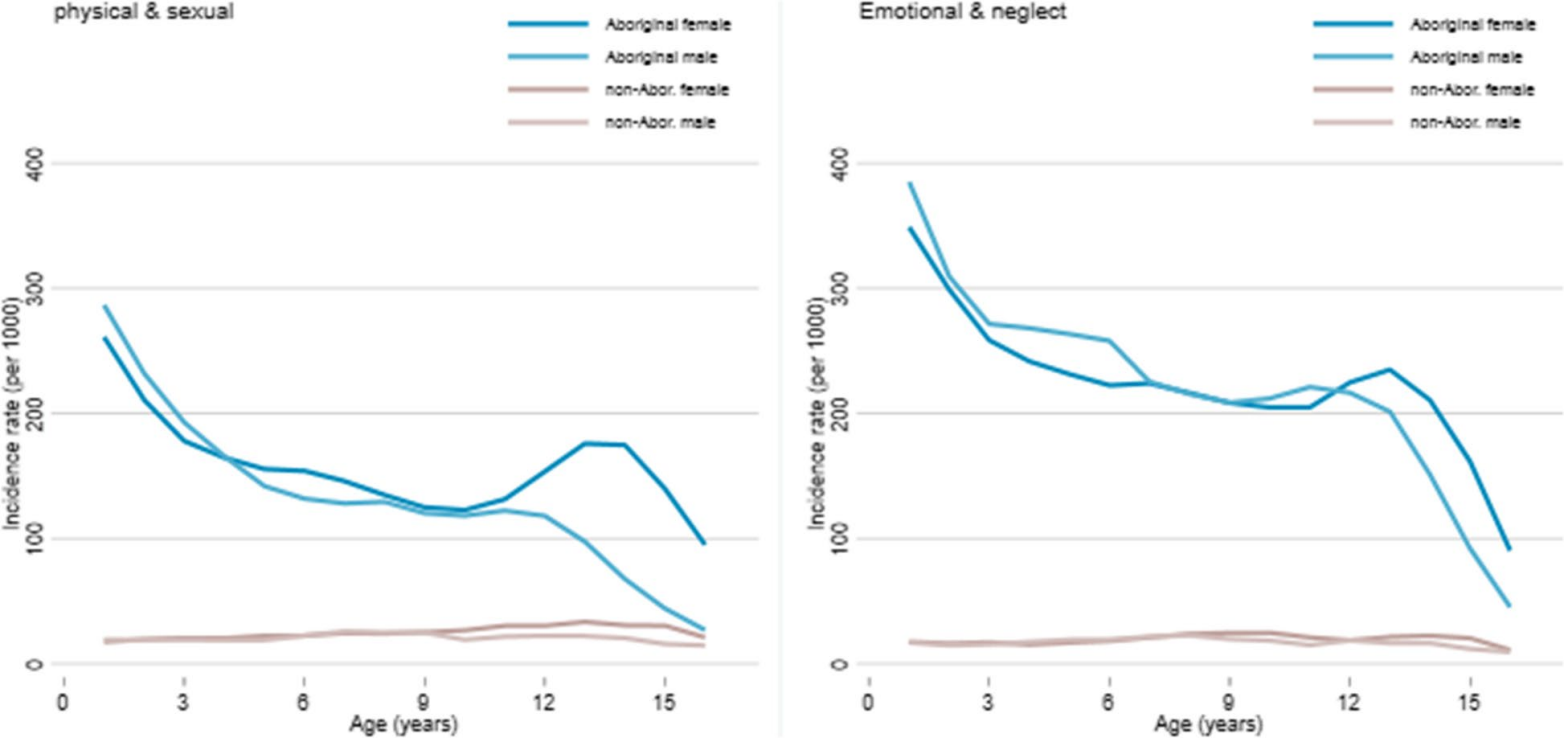
Incidence of substantiated maltreatment by year of age^1^ and type of abuse, NT 2011-2016. 1. Moving three-year age-groups from 0 to 2 years old to 15-17 years old. X-axis is the middle year of each three-year age group

A similar divergence between girls and boys during adolescence is apparent in the incidence of child maltreatment, particularly for physical and sexual abuse. The incidence of substantiated emotional abuse and neglect was similar for boys and girls until age 12 years after which there was a higher incidence for Aboriginal girls than boys (Fig. 2). For physical and sexual abuse, incidence was similar for girls and boys to about age 11, but higher for girls than boys thereafter. This divergence was greater among Aboriginal than non-Aboriginal children; the incidence of physical and sexual abuse was over three times higher for Aboriginal girls than boys aged 14-17 years but only 1.8 times higher for non-Aboriginal girls than boys (Table 4).

**Table 4:** Substantiated physical or sexual abuse incidence by age-group^a^, Northern Territory 2011-2016

Age-group	Aboriginal				Non-Aboriginal			
	Male	Female	Ratio^b^		Male	Female	Ratio^b^	
0-9	188.1	188.3	1.00	(0.93-1.08)	20.8	21.1	1.01	(0.84-1.22)
10-13	116.5	140.5	1.21	(1.03-1.40)	23.5	29.5	1.26	(0.96-1.64)
14-17	33.7	114.1	3.38	(2.68-4.26)	14.0	25.9	1.84	(1.32-2.57)

^a^ incidence rate per 1000

^b^ incidence rate ratio (female:male) and 95% confidence interval

### Cumulative incidence between twelfth and eighteenth birthday

For children who turned age twelve in 2006 to 2010, 4.6% of Aboriginal girls were hospitalized after an assault at least once between their twelfth and eighteenth birthday, a higher proportion than for Aboriginal boys and much higher than for non-Aboriginal boys or girls (for whom hospitalization after assault was rare) (Table 5). For non-Aboriginal children, hospitalization after assault was less common for girls than boys (hazard ratio 0.42, 95%CI 0.20-0.91) but for Aboriginal children more common for girls than boys (1.40, 1.07-1.75).

**Table 5:** Cumulative incidence (%, with 95% confidence interval) of hospitalization for any injury and injury caused by assault between twelfth and eighteenth birthday, NT children who turned age twelve in 2006-2010

	Aboriginal		Non-Aboriginal	
	Male *n* = 3355	Female *n* = 3268	Male *n* = 3667	Female *N* = 3241
*Injury (any) hospitalization*				
Cum. Incidence	17.3 (16.1-18.7)	10.9 (9.9-12.0)	11.8 (10.8-12.9)	6.7 (5.9-7.6)
> 1 episode^a^	26.6 (23.1-30.4)	29.3 (24.7-34.3)	22.2 (18.4-26.4)	22.2 (16.9-28.4)
*Assault hospitalization*				
Cum. incidence	3.4 (2.8-4.0)	4.6 (3.9-5.3)	0.7 (0.4-1.0)	0.3 (0.1-0.5)
> 1 episode	16.8 (10.4-25.0)	30.9 (23.6-39.0)	20.8 (7.1-42.2)	0.0 (0.0-33.6)
*Substantiated physical and/or sexual abuse*				
Cum. incidence	2.9 (2.3-3.5)	9.1 (8.2-10.2)	2.0 (1.6-2.5)	2.8 (2.3-3.5)
> 1 episode	14.6 (8.2-23.3)	21.4 (16.9-26.5)	19.4 (11.1-30.5)	18.5 (11.1-27.9)

^a^ proportion of children with any hospitalization/substantiation who had more than one hospitalization/substantiation

The proportion of children with any hospitalization for injury who had more than one such episode was higher for Aboriginal than non-Aboriginal children and similar for boys and girls. Thirty-one percent of Aboriginal girls who were hospitalized after assault had more than one such episode, almost double the proportion for Aboriginal boys.

The proportion of children who had one or more substantiated episodes of physical and/or sexual abuse between their twelfth and eighteenth birthdays was higher for Aboriginal than non-Aboriginal children (Table 5). Girls were more likely to have substantiated physical and/or sexual abuse than boys; this excess was greater for Aboriginal than non-Aboriginal girls (cumulative incidence ratio: Aboriginal 3.30, non-Aboriginal 1.45). The proportion of children with any substantiated episode of physical and/or sexual abuse who had more than one such episode was similar for all groups.

### Is serious assault more common for maltreated girls?

Analysis of the association between level of child protection involvement before age 12, and hospitalization after assault was restricted to Aboriginal children because there was an insufficient number of non-Aboriginal children hospitalized after assault (eight children, most of whom had no interaction with child protection services) to undertake statistical analysis of this relationship.

The incidence of injury hospitalization was greater for Aboriginal children with prior child protection interaction than those with no such interaction and increased with level of interaction (Table 6). There was suggestive evidence that the increase with level of child protection interaction was greater for girls than boys (the hazard ratio for the interaction term sex*CP level was 1.13, 95%CI 0.98-1.31) but the 95% confidence interval included 1.0 so the interaction term was not included in the final model.

**Table 6:** Multivariable analysis of the incidence of injury and assault hospitalization during adolescence^b^ by level of child protection contact before age twelve, Aboriginal children born in the NT in 1999-2003

	Any injury^b^		Assault^c^	
	Hazard ratio^a^ (95%CI)		Hazard ratio (95%CI)	
*Level of child protection involvement*				
None (reference)	1.00		1.00	
Notification only	1.38	(1.14-1.67)	1.73	(1.08-2.79)
Substantiation only	1.71	(1.38-2.11)	3.54	(2.26-5.56)
Out-of-home-care	1.97	(1.52-2.55)	3.56	(2.04-6.20)
Female^d^	0.62	(0.53-0.72)	2.29	(1.57-3.34)

^a^.the ratio of the incidence rate for children at each level of child protection interaction to those with no child protection interaction

^b^ first hospitalization for treatment of any injury between 12th and 18th birthday

^c^ first hospitalization for treatment of injury caused by assault between 12th and 18th birthday

^d^ compared with male

For assault, the incidence of hospitalization was also greater for children with a record of prior child protection involvement than those with no record of child protection involvement. This difference was particularly evident for those children with a record of substantiated abuse (whether or not in out-of-home-care), for whom the incidence of assault hospitalization was 3-4 times higher than for children with no child protection involvement (Table 6). There was no evidence that the association between level of child protection involvement and incidence of assault hospitalization was different for girls than boys (the hazard ratio for the interaction term sex*CP level was 0.99, 95%CI 0.71-1.38).

## Discussion

This study demonstrates that the elevated risk of violence against Aboriginal women is not restricted to adults but commences around puberty and accelerates through adolescence. By the age of 14-17 years the risk of hospital admission for assault for Aboriginal girls is twice the corresponding rate for Aboriginal boys and more than 30 times greater than non-Aboriginal girls of the same age.

Dysfunctions within family, peer groups, intimate partners and the communities in which adolescent Aboriginal girls live contribute in diverse and inter-related ways to the very high level of violence they experience. Underlying these intermediary factors is the on-going damage to Aboriginal families, culture and society resulting from the invasion and colonization of their land 234 years ago. In Aboriginal society, protection within families and communities is supported by the positive influence of cultural factors such as caring for country, knowledge and beliefs, language, kinship responsibilities and cultural expression [20, 21]. All of these have been severely damaged over many generations by the racial discrimination and cultural genocide that colonization brought to Aboriginal Australians, as to First Nations peoples worldwide [22]..

### Family violence: direct and indirect effects

Family violence is considerably more common for Aboriginal than other Australians [23]. Family violence contributes directly to the very high levels of violence inflicted on Aboriginal girls during adolescence, as is apparent in their high incidence of physical and sexual maltreatment and the high proportion of family members (usually not parents) among perpetrators of assault requiring hospitalization. Family dysfunction and violence also contributes indirectly, in several ways. This study found that maltreatment during childhood increases the risk of serious assault during adolescence. This overlap of risk is unsurprising. The extended family and the broader community environment play an important role in keeping children and adolescents safe. Drug and alcohol abuse, mental illness and domestic violence within the family increase the risk of child maltreatment and compound the child’s continuing vulnerability into adolescence. If the environment remains unchanged as the child moves through to adulthood, the increased risk remains. Additionally, traumatic childhood experience of disrespect, abuse and violence (including observing that experienced by other family members) can normalize these behaviors as part of peer, partner and parenting relationships [24]. Abusive and violent behavior is thus perpetuated from generation to generation.

### Peer groups, identity and independence

Violence towards adolescent Aboriginal girls may occur in the context of complex peer group dynamics as part of the passage to adulthood. As noted in a study of a remote Northern Territory community, young women engage in behaviors of ‘walking about at night’ [25] as a defining feature of adolescence, with the result often being pregnancy. The study examined participants’ perceptions of their agency and sense of control within these behaviors; relationships and pregnancy during adolescence linked to their identity. Where young women are powerless in the relationship, or taught to be submissive and inferior to men, they are at risk of coercive behavior and violence, both physical and sexual. Dangerous family and community circumstances can lead to adolescent girls seeking a level of compromised independence, the compromise being to their own safety. Adolescent girls may remove themselves from dangerous family or community circumstances, seeking independence and connection through relationships with their peers, that also may involve a level of risk [20].

### Intimate partner violence and exploitation

Parents and carers are the most commonly recorded perpetrator for younger Aboriginal children; this pattern shifts through adolescence and for 14-17 year old girls the most commonly reported perpetrators (40%) are their partners. Violence can also come from boys or men who engage adolescent girls in exploitative transactional sexual activity through payment of money, alcohol or drugs. Through such rewards, some girls seek power and independence. Examples from the research include where girls are aware of the risks of violence within intimate partner relationships that include coercive control of their behaviors, the way they dress and the violence surrounding jealousy by their partner [21]. The agency of adolescent girls within such consensual or exploitive relationships suggests awareness of the dynamics yet only limited practical strategies for remaining safe in circumstances that are realistically outside their control. Some girls report the preference to remain single and allow the boys to fight between themselves rather than with their partner.

### Gaps in systems and responses

Aside from the initial health response, the primary agencies tasked with responding to children who have been physically or sexually harmed are child protection and police. However, a response to the harm of adolescent girls is a grey area of policy and practice across both agencies and there are many scenarios suggestive of harm that do not meet each agency’s threshold for intervention. The response from child protection services focuses on harm caused by family members, or through the inability of parents to protect the child from someone within or outside the family. The child protection service would not usually respond to harm caused by someone outside the family such as the young person’s partner (unless the adolescent girl is also the mother of a child considered in danger of harm or neglect). Police responses are limited by the ‘investigative’ nature of policing; the capacity of police to protect adolescent girls is limited if the details of the perpetrator are not disclosed. There are many complex reasons why the perpetrator of violence is not disclosed, even when police or medical responses are initially required. The silencing and silence of Aboriginal girls can serve to prohibit the involvement of child protection and criminal justice responders, rendering the investigatory-oriented system ineffective in tackling the problem of violence towards adolescent Aboriginal girls.

The NT government’s ‘Domestic, Family and Sexual Violence Reduction Framework, 2018-2028’ [4] focuses on the high level of violence inflicted on Aboriginal women and children and the need for a multi-faceted approach to prevention and protection, but it does not recognize the particularly parlous situation of many adolescent girls and their specific circumstances and needs. The findings of this study present an opportunity to recognize gaps in existing policy and services to anticipate and mitigate the violence experienced by adolescent Aboriginal girls that is perpetrated by those outside family, within family, within intimate partner relationships, throughout pregnancy and as young mothers.

Within the NT the reporting to police of imminent violence by a partner is already mandated for all Territorians, as it is for child abuse. However, there is no clear pathway of support or referral where Aboriginal adolescent girls can proactively seek support to be kept safe from violent partners, other than through a Domestic Violence Order. Although there remains a role for investigative responses by police and child protection authorities to violence against children when they are young or through adolescence, a more sophisticated and comprehensive approach would ensure the assessment of danger, the engagement of the perpetrator of violence and the early connection of appropriate networked supports for each.

### Role of Aboriginal culture in system response

Critical in framing the response is the recognition of culture. The Territory Families, Housing and Communities’ *Aboriginal Cultural Security Framework* [13] sets the benchmark for individual and agency-wide practice for engaging Aboriginal young people at risk and their families. One small study involving Aboriginal women through pregnancy [26] identified the need for assuring cultural safety, being non-judgmental, and providing support and validation when inquiring about intimate partner violence. Another recent study identified the disempowered and silenced voices of Aboriginal women that resulted in the tolerance of increased levels of violence [27]..

While better systems are needed to improve the safety of adolescent girls and reduce the recurrence of violence after it occurs, improving the capacity and function of Aboriginal families and society is the long-term solution to this problem, as it is for so many of the health, social and economic issues disproportionately afflicting Aboriginal Australians and other First Nations peoples. For community approaches, it can be reasonably argued that investigative responses to the physical and sexual harm caused to young children and adolescents can only play a small part in addressing a wider social phenomenon but that it can be a component part of community-led and supported strategies for tackling violence. For example, a study of community-involved primary prevention of family violence in regional Western Australia [28] recognized the factors that impact community readiness for preventive intervention, and that such change would necessarily be intergenerational and could be supported by greater community-wide education on the gendered causes of family violence.

### Strengths and limitations

There are strengths and limitations to this study. A strength is that population-level linked data provides a comprehensive coverage and representativeness of the study population. The study also allows appropriate comparison between the four groups reported. There are also limitations. As described earlier in the paper, while hospital admission data provides a reliable record for serious assault, admissions are only a small proportion of all assault events. Victims of assault-related injuries may be treated in emergency departments or in primary care clinics, while many others do not access any medical care. A second limitation is that while the use of linked administrative data provides an important but uncommon insight into the vulnerability of adolescent Aboriginal girls, administrative data does not capture critical social and cultural dimensions of Aboriginal girls’ experiences that may benefit from a mixed methods approach. Such an approach would also allow the collection of information on modifiable risk and protective factors, including from the girls themselves, that would illuminate opportunities for preventive intervention.

## Conclusion

The study highlights the vulnerability of Aboriginal adolescent girls to physical and sexual harm and is a catalyst for the consideration of the adequacy of existing law, policy and practice in attending to the problem. Child protection systems can be blind to the incidence of violence towards adolescent Aboriginal girls where the harm does not occur within the family context, but rather with similar age partner or peer group. Similarly, where adolescent girls do not disclose the perpetrator of physical or sexual harm, the protections afforded by the criminal justice system are not available to them. Domestic violence intervention and support focuses on adults within violent relationships, rather than those under 18. With the findings of this study, opportunity presents for examining policies and strategies for the reduction of domestic and sexual violence. In doing so, it will be important to include the community and young people in identifying the most appropriate strategies to support the reduction of such violence.

## Data Availability

The study datasets contain sensitive personal information and are held on a secure cloud-based server with restricted access. Access requires the approval of the ethics committee and data custodians. For applications for data access, please contact the Menzies Data-linkage Program Leader at steve.guthridge@menzies.edu.au. We have ethics approval for this project (HREC-2016-2708) as previously stated.
